# Intraoperative Bowel Decompression Using an Endo GIA-to-Ultrasound Sleeve Anastomosis: A Novel Approach for Managing Unprepped Sigmoid Volvulus

**DOI:** 10.7759/cureus.86970

**Published:** 2025-06-29

**Authors:** Brandt Gruizinga, Jonathan Santos, Kevin Szafran, Daniel De Oliveira

**Affiliations:** 1 General Surgery, Nassau University Medical Center, East Meadow, NY, USA; 2 Medical Education, American University of the Caribbean School of Medicine, East Meadow, NY, USA; 3 Thoracic Surgery, Nassau University Medical Center, East Meadow, NY, USA

**Keywords:** bowel obstruction, colorectal surgery, endo gia, fecal decompression, intraoperative technique, sigmoid volvulus, stapled anastomosis, surgical innovation, ultrasound sleeve, unprepped colon

## Abstract

Acute large bowel obstruction from sigmoid volvulus in unprepped patients presents a significant intraoperative challenge due to the high risk of contamination when decompressing or resecting a distended, feces-filled colon. This paper describes a novel technique for safe and effective decompression of the colon using a linear GIA stapler to create an anastomosis between the descending colon and a sterile ultrasound (US) probe sleeve, enabling fecal evacuation without contamination of the operative field. A case report is presented, and the potential for this technique to be validated through future animal studies is discussed.

## Introduction

Sigmoid volvulus is a common cause of large bowel obstruction in elderly populations. In the United States and Western Europe, it accounts for approximately 10-15% of large bowel obstructions. In resource-limited regions, such as parts of Africa, South America, and Asia, it can represent up to 42% of cases [1]. Sigmoid volvulus frequently requires emergency surgical intervention when nonoperative decompression fails or when ischemia or perforation is suspected [2]. In unprepped patients, intraoperative management presents a particular challenge due to the increased risk of fecal contamination during decompression or resection.

Traditional decompression techniques such as manual evacuation, on-table lavage, or enterotomy with suction carry substantial risks of intraoperative spillage and postoperative infectious complications. Surgical site infection (SSI) rates following colorectal surgery range from 5% to 30%, with rates as high as 80% in cases involving perforation [3]. Beyond infection, fecal contamination contributes to significant peritoneal inflammation, which can lead to the development of intra-abdominal adhesions.

Adhesions form in more than 50% of patients following intra-abdominal surgery and are driven by peritoneal trauma and inflammation [4]. Notably, fecal spillage is a major aggravating factor in adhesion formation, as shown by increased rates of adhesion-related readmissions following colorectal procedures [5]. These adhesions can lead to chronic pain, infertility, and recurrent bowel obstructions, creating a self-perpetuating cycle of inflammation, reoperation, and further adhesion formation. Avoiding the initial inflammatory insult of fecal contamination therefore offers the potential for unquantifiable cost savings and improved long-term patient outcomes.

Preserving a sterile operative field is thus essential but technically difficult in the setting of a massively distended colon. In this report, we describe a novel approach using an Endo GIA stapler to create a sealed, side-to-side anastomosis between the descending colon and a sterile ultrasound (US) probe sleeve. This configuration allows for controlled fecal evacuation into a closed system, reducing intraoperative contamination and preserving sterility.

By repurposing familiar surgical tools in an innovative manner, this technique may serve as a safer, reproducible alternative for decompression in emergent colorectal procedures. Further validation through animal models and clinical studies is warranted to assess safety, feasibility, and broader applicability.

## Case presentation

Patient information

A male patient in his mid-60s with baseline cognitive impairment and residing in an assisted living facility presented to the emergency department with nausea and vomiting. While his symptoms were initially nonspecific, physical examination revealed a distended, tympanic abdomen with mild diffuse tenderness and no signs of peritonitis. He was hemodynamically stable. A contrast-enhanced CT scan demonstrated a markedly dilated sigmoid colon with mesenteric twisting, consistent with sigmoid volvulus.

Following fluid resuscitation, electrolyte correction, and nasogastric decompression, the decision was made to proceed with an exploratory laparotomy due to concern for complete colonic obstruction. Intraoperatively, a massively distended sigmoid colon with volvulus was confirmed. Given the absence of preoperative bowel preparation, controlled decompression was prioritized to minimize contamination and allow safe resection.

Intraoperative findings

**Figure 1:**
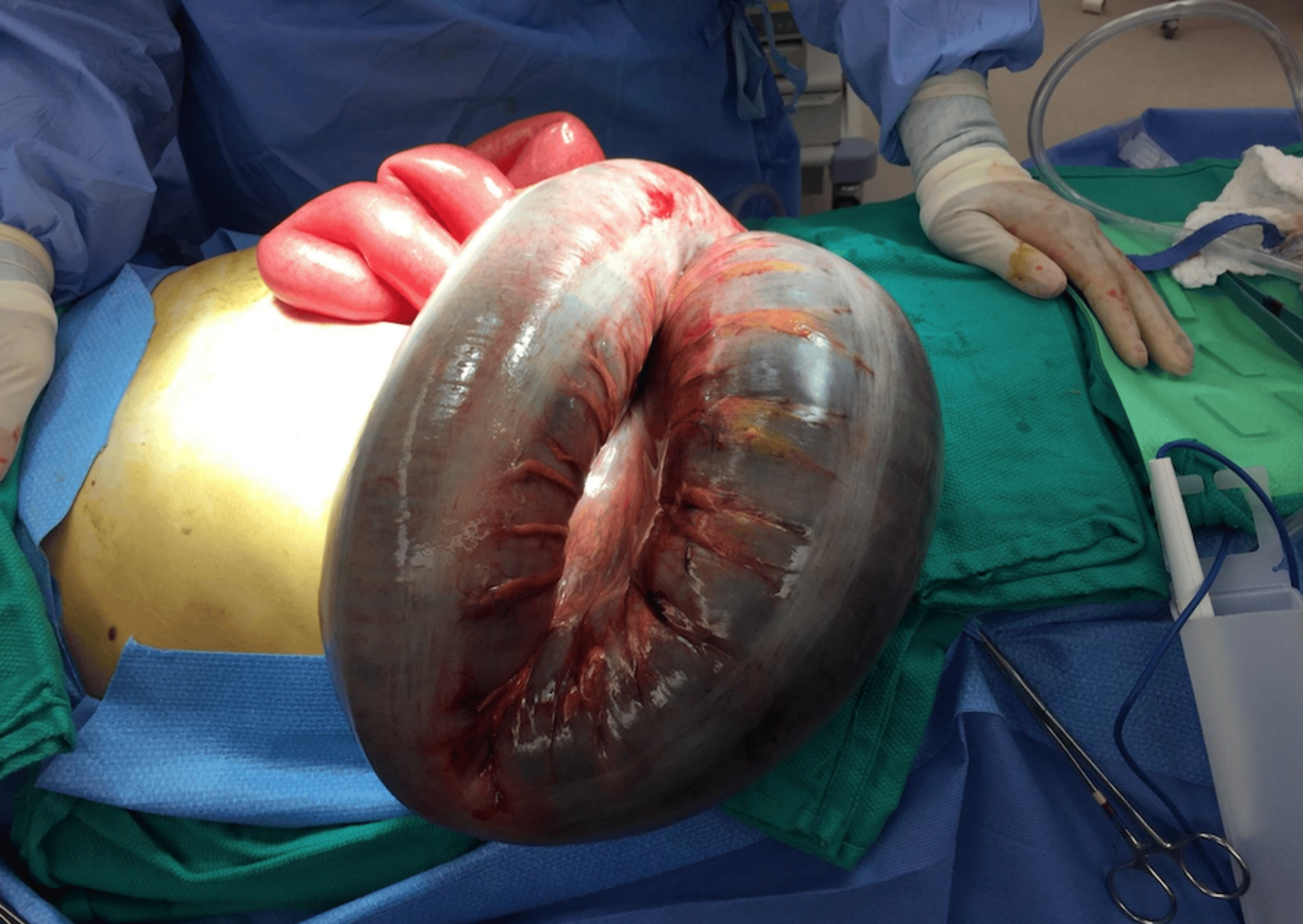
Intraoperative image following a midline laparotomy, showing a grossly distended sigmoid colon with severe dilation. No evidence of ischemia or perforation is observed.

A midline laparotomy revealed a grossly distended sigmoid colon. There was no evidence of ischemia or perforation. The bowel was severely dilated (up to 12 cm in diameter) with thin bowel walls, and the descending colon proximal to the twist was moderately dilated (Figure 1).

Decompression technique

**Figure 2:**
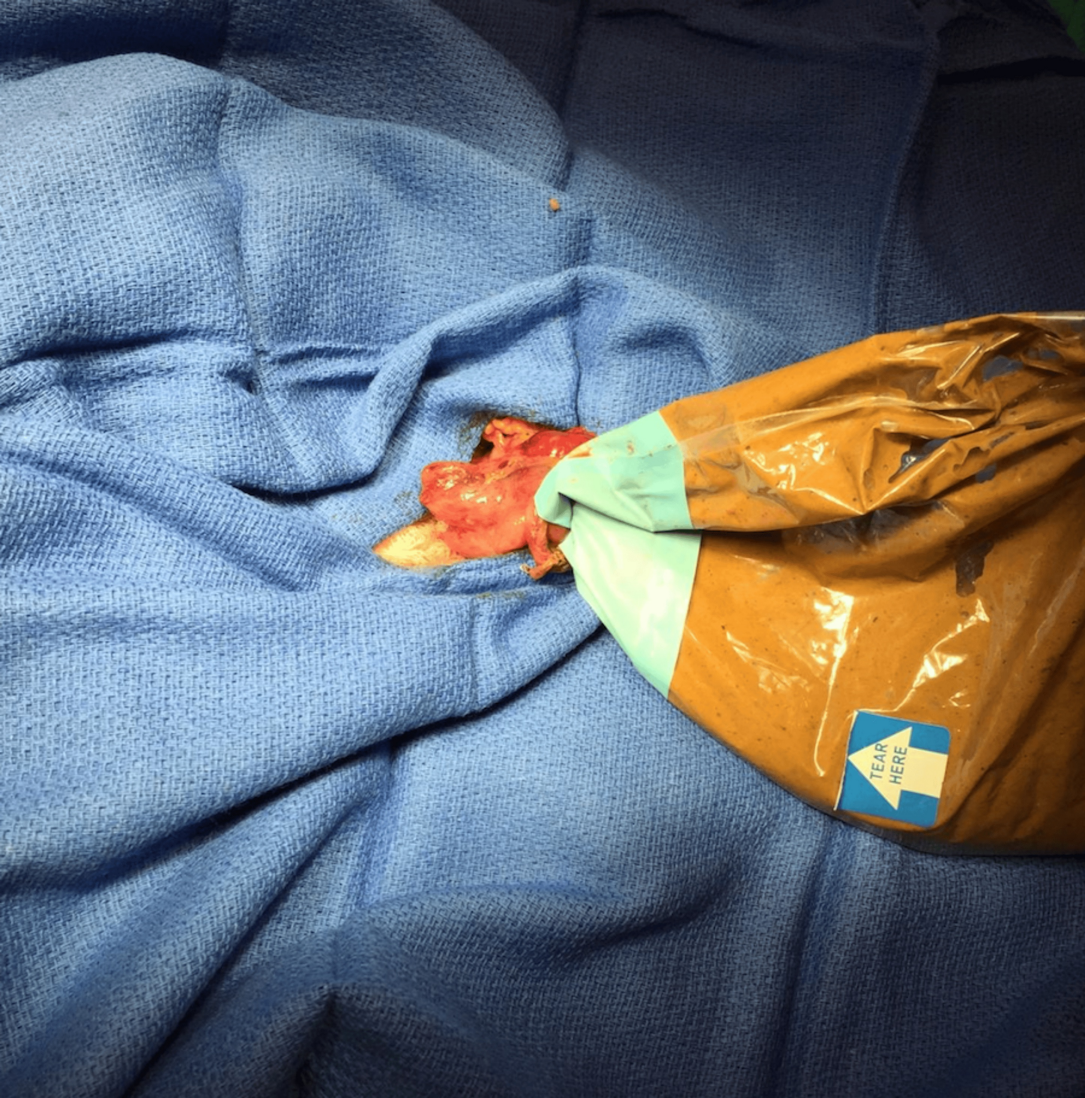
Intraoperative image showing a side-to-side anastomosis made between the descending colon and a sterile US probe sleeve US: Ultrasound

Given the lack of preoperative bowel preparation and the high risk of fecal spillage, decompression in a controlled manner to minimize spillage was paramount. The sigmoid colon was transected distal to the point of torsion using a linear 60 mm Endo GIA purple-load stapler. A second linear stapled side-to-side anastomosis was created between the proximal descending colon and a sterile US probe sleeve, which had been modified and clamped distally to act as a closed fecal conduit (Figure 2).

**Figure 3:**
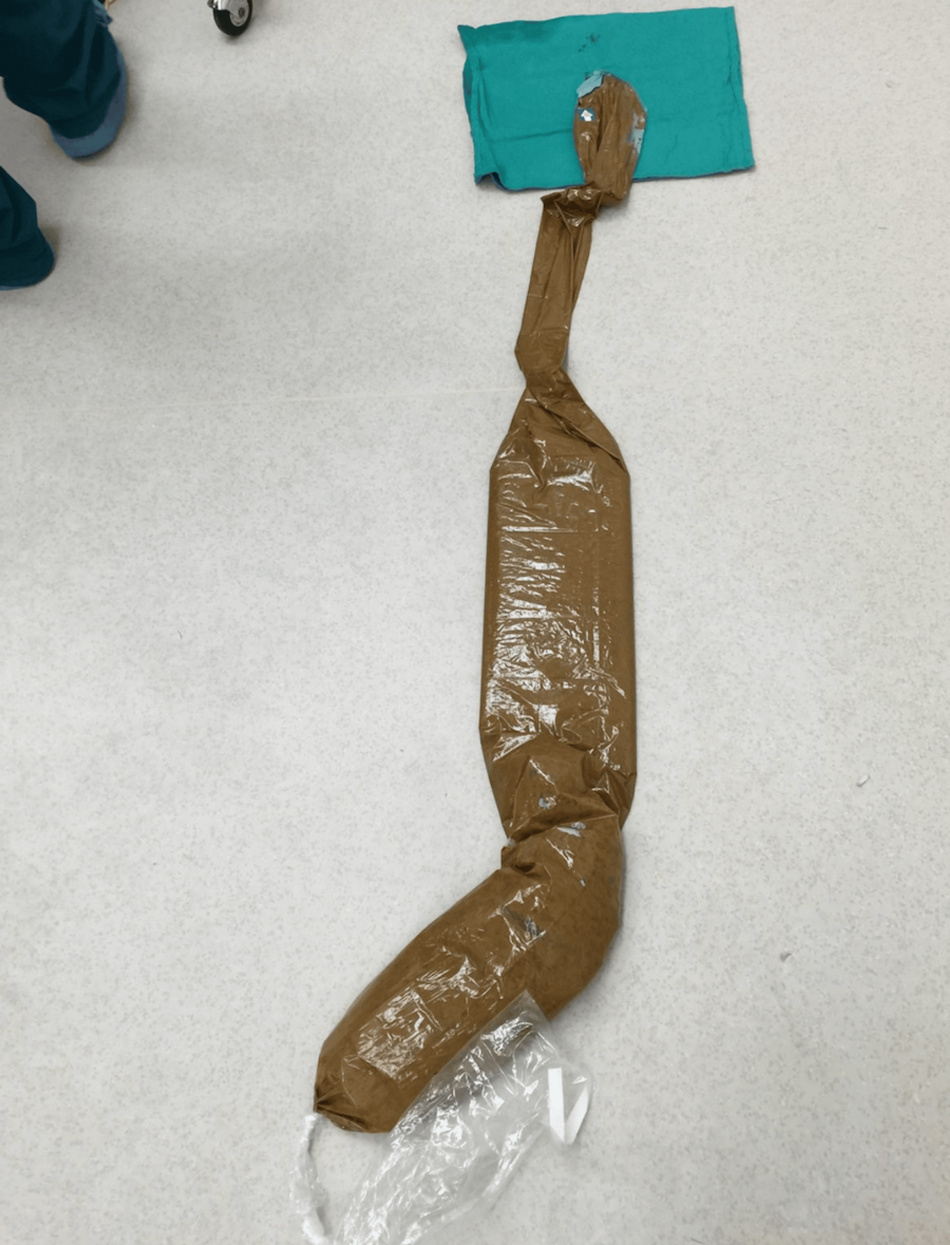
The sterile US sleeve filled with approximately 2 liters of fecal matter after being removed from the operative field US: Ultrasound

Once the stapler fired, immediate decompression was achieved as fecal content flowed into the contained sleeve. Approximately 2 liters of semisolid fecal material was evacuated without contamination of the surgical field. The sleeve was clamped proximally and removed from the field in one motion, preserving sterility (Figure 3).

Definitive surgery and colostomy

After complete decompression, the remaining redundant sigmoid colon was resected. A formal end colostomy was constructed in the left lower quadrant. The rectal stump was oversewn and placed in the pelvis. The colostomy was matured with attention to creating a tension-free stoma and securing the mesentery. No intraoperative complications occurred. 

Postoperative course and follow-up

The patient had an uneventful postoperative course. He was advanced from clear liquids to a soft diet by postoperative day (POD) 3, with return of bowel function by POD 4. Pain was well controlled with oral analgesics, and he was ambulating independently by POD 2. Laboratory markers remained within normal limits, and no signs of infection or anastomotic complications were observed.

The patient was discharged in stable condition on POD 6 with colostomy education and outpatient follow-up arranged. At his two-week postoperative visit, he reported no complications, and his surgical wounds were healing well.

## Discussion

Intraoperative decompression of a distended, unprepped colon remains a major challenge in emergency colorectal surgery. Conventional methods such as manual decompression, suction via enterotomy, or on-table lavage have been associated with increased rates of fecal contamination, which can elevate the risk of postoperative infectious complications, including wound infections and intra-abdominal abscesses, and lead to prolonged recovery and hospital stay [2].

The technique described here offers a novel, controlled approach by creating a side-to-side stapled anastomosis between the colon and a sterile US sleeve. This closed conduit allows for safe evacuation of fecal contents without contaminating the operative field. It uses standard surgical tools in an unconventional but practical way, making it easily adoptable across a range of surgical settings.

The implications of fecal spillage extend beyond infection alone. Contamination can also contribute to the formation of intra-abdominal adhesions, which have been linked to chronic pain, infertility, and future bowel obstructions. A systematic review by ten Broek et al. identified postoperative contamination as a contributing factor to adhesion-related morbidity [6]. More recently, Krielen et al. showed that contamination significantly increases the risk of adhesion-related readmissions following colorectal surgery, reinforcing the long-term burden of even a single intraoperative contamination event [5].

Maintaining sterility is critical, especially in unprepped patients, to reduce complications such as wound infections, intra-abdominal abscesses, and adhesions. This method may also be particularly valuable in resource-limited or high-risk environments, where bowel preparation is not feasible and contamination control is essential. Further studies, including animal models, are warranted to evaluate reproducibility, safety, and broader applications in emergency colorectal care.

## Conclusions

Intraoperative bowel decompression using an Endo GIA-to-US sleeve anastomosis offers a safe, sterile, and effective solution for managing unprepped sigmoid volvulus. By minimizing contamination and simplifying fecal evacuation, this technique addresses a critical challenge in emergency colorectal surgery. Its ease of adoption and potential to reduce postoperative complications position it as a promising alternative in high-risk and resource-limited settings.
